# Duchenne Muscular Dystrophy: Integrating Current Clinical Practice with Future Therapeutic and Diagnostic Horizons

**DOI:** 10.3390/ijms26146742

**Published:** 2025-07-14

**Authors:** Costanza Montagna, Emiliano Maiani, Luisa Pieroni, Silvia Consalvi

**Affiliations:** 1 Departmental Faculty of Medicine, UniCamillus—Saint Camillus International University of Health and Medical Sciences, 00131 Rome, Italy; costanza.montagna@unicamillus.org (C.M.); emiliano.maiani@unicamillus.org (E.M.); luisa.pieroni@unicamillus.org (L.P.); 2 IRCCS San Camillo Hospital, 30126 Venice, Italy

**Keywords:** Duchenne muscular dystrophy, antisense oligonucleotides, gene therapy, HDAC inhibitors, CRISPR-Cas systems, genetic testing, early diagnosis

## Abstract

Duchenne muscular dystrophy (DMD) is a severe X-linked disorder characterized by progressive muscle degeneration due to mutations in the dystrophin gene. Despite major advancements in understanding its pathophysiology, there is still no curative treatment. This review provides an up-to-date overview of current and emerging therapeutic approaches—including antisense oligonucleotides, gene therapy, gene editing, corticosteroids, and histone deacetylases(HDAC) inhibitors—aimed at restoring dystrophin expression or mitigating disease progression. Special emphasis is placed on the importance of early diagnosis, the utility of genetic screening, and the innovations in pre-and post-natal testing. As the field advances toward personalized medicine, the integration of precision therapies with cutting-edge diagnostic technologies promises to improve both prognosis and quality of life for individuals with DMD.

## 1. Duchenne Muscular Dystrophy: Epidemiology and Pathophysiology

Duchenne muscular dystrophy (DMD) is a severe, progressive condition characterized by muscle degeneration, with early signs including difficulty in walking and frequent falls beginning around the ages of 2 to 3 [1,2]. By the age of 10 to 12, most individuals require a wheelchair, and by 20, many will need mechanical ventilation. Life expectancy typically ranges from 20 to 40 years, depending on population, access to healthcare, cohort of birth, and availability of multidisciplinary care [1,2]. Before the 1990s, most patients lived only into their late teens or early twenties. Since then, advances like corticosteroids, ventilation, and cardiac care have extended survival into the late twenties. In high-income countries, many born after 2000 now live into their thirties or forties, while in lower-income regions, limited care often keeps life expectancy below the mid-twenties [3].

DMD is caused by mutations in the DMD gene, which encodes dystrophin (DMD phenotype: MIM #310200; Gene/Locus dystrophin: *MIM 300377). These mutations prevent the production of functional dystrophin, a crucial protein for muscle fibers integrity. Frameshift or nonsense mutations result in truncated, non-functional dystrophin. A related, milder disease—Becker muscular dystrophy (BMD)—is caused by mutations that preserve the open reading frame, producing a partially functional dystrophin protein [1,2]. The prevalence of Duchenne muscular dystrophy (DMD) is approximately 1 in 3500 to 5000 male births. As for Becker muscular dystrophy (BMD), its prevalence is about 1 in 18,000 to 30,000 male births. Both conditions are X-linked recessive and predominantly affect males. Females who carry the mutated gene typically show no symptoms, although some (8% to 10%) may experience mild muscle weakness or heart issues. Approximately 60–70% of DMD and BMD cases are due to gene deletions, though other mutations, such as duplications and point mutations, are also responsible. About one-third of DMD cases are caused by new mutations [1,2,4].

Dystrophin works by forming the dystrophin-associated protein complex (DAPC), which links the cytoskeleton inside muscle cells to the extracellular matrix and supports muscle stability [5]. The DAPC includes dystrophin and several proteins such as dystroglycans, sarcoglycans, syntrophins, dystrobrevins, and neuronal nitric oxide synthase (nNOS). Dystrophin interacts with actin, microtubules, intermediate filaments, and many signaling and scaffolding proteins through multiple binding domains [5,6]. Through its network of proteins, DAPC maintains muscle cell structure, supports signaling pathways, and contributes to overall muscle function [5,6,7].

The absence of dystrophin causes the disassembly of the dystrophin-associated protein complex (DAPC), disrupting the link between the cytoskeleton and the extracellular matrix and leading to various functional impairments in muscle cells [8,9,10,11,12,13,14,15]. Without the DAPC, the sarcolemma becomes fragile and prone to damage during contractions. This fragility results in membrane tears, leakage of enzymes such as creatine kinase (CK), and increased susceptibility to damage in heavily used muscles, like the diaphragm [9].

Dystrophin and the components of the DAPC act as molecular scaffolds that organize various signaling molecules, including ion channels. These elements are essential for maintaining ion homeostasis, particularly calcium balance, which is crucial for proper muscle contraction and relaxation. Loss of dystrophin disrupts the function of ion channels in the sarcolemma and intracellular organelles such as the sarcoplasmic reticulum and mitochondria. This affects the regulation of calcium, sodium, and potassium ions, leading to impaired muscle function and contributing to the progression of muscle pathology [10]. Targeting these ion channels and correcting the underlying ionic imbalances may offer effective therapeutic strategies.

Dystrophin also normally anchors neuronal nitric oxide synthase (nNOS) to the membrane, promoting blood flow during exercise. In its absence, this mechanism fails, leading to poor blood supply and ischemic muscle damage [11]. Mislocalized nNOS and malfunctioning mitochondria contribute to the excessive production of reactive oxygen and nitrogen species. This causes oxidative damage to proteins, lipids, and DNA, while antioxidant defenses, like glutathione, are diminished [12]. Defective proteins and organelles accumulate and are not efficiently removed by autophagy, which is severely repressed in DMD [13]. Damaged muscle attracts immune cells such as macrophages and T cells, which initially attempt to repair the tissue but eventually contribute to fibrosis and fat infiltration due to persistent inflammation and elevated TGFβ levels [14]. The disassembly of the DAPC also directly affects muscle regeneration, impairing satellite cell function and division, which are essential for muscle repair [15].

Overall, the combined effects of mechanical weakness, ion dysregulation, poor blood supply, oxidative stress, chronic inflammation, and impaired regeneration drive the progressive muscle degeneration and replacement by fat and fibrotic tissue that characterize DMD.

## 2. Current and Emerging Treatments

**Figure 1:**
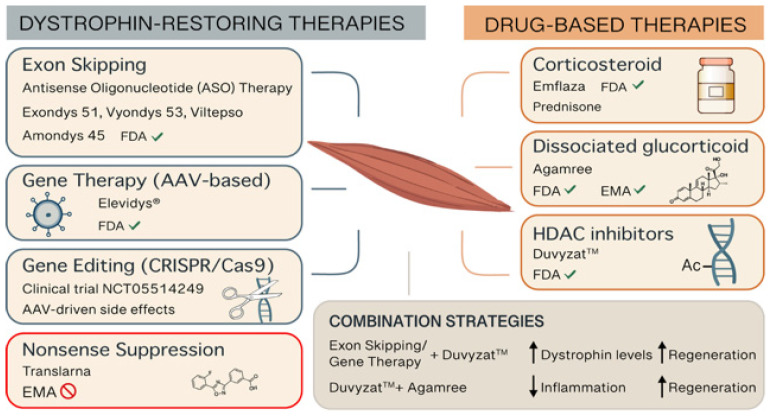
Representative picture of the therapeutic options for DMD. Abbreviations: AAV (adeno-associated virus), CRISPR (Clustered Regularly Interspaced Short Palindromic Repeats), EMA (European Medicines Agency), FDA (Food and Drug Administration), HDAC (histone deacetylase).

Despite significant therapeutic advances over the past 30 years, there is still no cure for DMD. However, a multidisciplinary medical, surgical, and rehabilitative approach can improve quality of life and increase longevity for patients [16]. In the last 20 years, various therapeutic strategies have been developed to address different aspects of DMD pathophysiology. These strategies generally fall into two main categories: those aimed at restoring dystrophin production and those focused on mitigating the secondary effects caused by the lack of dystrophin (Figure 1). Many of these treatments are currently under investigation in clinical trials and are not yet available for routine clinical use. However, a few of these therapies have already received regulatory approval by U.S. Food and Drug Administration (FDA) and the European Medicines Agency (EMA) (Table 1).

### 2.1. Corticosteroid Therapy

Corticosteroids are the current standard treatment for DMD, designed to manage symptoms and delay disease progression by exerting strong anti-inflammatory effects. Current guidelines recommend the use of the glucocorticoids prednisone or deflazacort in boys with DMD when motor development stops or begins to decline, with treatment continued throughout life. Therapy is usually initiated around 4–5 years of age, but not before the age of 2 [17]. Both drugs show similar benefits in improving strength and motor function, delaying loss of ambulation, preserving pulmonary function, reducing the need for scoliosis surgery, and postponing the onset of cardiomyopathy. Some studies suggest that deflazacort may have an advantage over prednisone in delaying loss of ambulation and increasing survival, but the evidence remains controversial [17,18,19]. Although only deflazacort is FDA approved for DMD treatment, both prednisone and deflazacort are used as the standard of care. Nevertheless, glucocorticoid treatment is also associated with side effects linked to mineralocorticoid activity, such as hypertension, fluid retention, weight gain, skin atrophy, and bone loss [17]. Other common adverse effects include cushingoid appearance, hirsutism, growth delay, irritability, skin thinning, and easy bruising. Additionally, long-term use of deflazacort may be associated with cataracts.

Currently, an alternative drug, Vamorolone may reduce these side effects thanks to its antagonistic activity on mineralocorticoid receptors. Vamorolone (AGAMREE^®^) is a first-in-class, oral, selective, dissociative steroidal anti-inflammatory drug developed by ReveraGen BioPharma (Rockville, MD 20850-USA) and Santhera Pharmaceuticals (4133 Pratteln | Switzerland-EU) for the treatment of patients with DMD. Acting as a dissociative agonist of the glucocorticoid receptor, Vamorolone exerts anti-inflammatory and immunosuppressive effects while demonstrating in clinical trials similar efficacy and reduced safety concerns compared to conventional corticosteroids [20,21]. Based on these clinical benefits, FDA approved Vamorolone in October 2023 for the treatment of DMD in patients aged 2 years and older. In the same month, EMA issued a positive opinion recommending its approval for patients aged 4 years and older. The European Commission subsequently granted marketing authorization for AGAMREE^®^, making it the only approved medication for DMD in the European Union and the first DMD treatment approved in both the U.S. and EU [22].

### 2.2. Nonsense Suppression Therapy

Ataluren (also known as PTC124 and marketed as Translarna by PTC Therapeutics) is an orally bioavailable small molecule designed to treat DMD caused by nonsense mutations by inducing ribosomal readthrough, allowing production of functional dystrophin protein. Ataluren is used to treat patients aged 2 years and older who are able to walk. Although initial randomized, placebo-controlled trials showed trends of efficacy—such as modest improvements in walking distance and timed function tests—they failed to meet primary endpoints, leading to conditional approval by EMA in 2014 for ambulatory patients aged 2 years and older [23,24,25,26]. No FDA approval was ever granted.

However, subsequent post-marketing studies aimed at confirming efficacy did not demonstrate statistically significant benefits compared to placebo. Real-world data from patient registries also failed to provide conclusive evidence due to methodological limitations and biases [24]. After multiple re-examinations and evaluations—including consideration of patient and caregiver perspectives—the EMA’s Committee for Medicinal Products for Human Use (CHMP) concluded in 2024 that the effectiveness of Translarna could not be confirmed. As a result, on March 2025, the European Commission decided not to renew the conditional marketing authorization, and Translarna is no longer approved in the EU [27].

### 2.3. Antisense Oligonucleotide Therapy

Due to the large size of the dystrophin gene, which spans 79 exons, it is highly susceptible to various types of mutations—most commonly intragenic deletions, particularly within the exon 45–53 “hotspot” region. These mutations often result in a frameshift, yielding a nonfunctional or absent dystrophin protein [1,2].

Among the several strategies developed to treat DMD, exon skipping via antisense oligonucleotide (ASO) therapy has emerged as one of the most promising mutation-specific approaches. ASOs are short, synthetic nucleic acid sequences designed to modulate pre-mRNA splicing. They bind via base-pairing to specific exonic or intronic regions of the pre-mRNA, causing the spliceosome to skip over a targeted exon. This can restore the open reading frame and allow the production of a truncated but functional dystrophin protein, resembling the milder BMD phenotype [28,29].

The most clinically advanced ASOs for DMD are based on phosphorodiamidate morpholino oligomer (PMO) chemistry. PMOs are synthetic molecules that resist degradation by nucleases, exhibit favorable safety profiles, and are not immunogenic. Currently, four PMO-based ASOs have been granted accelerated approval by the U.S. Food and Drug Administration (FDA) for treating specific DMD genotypes: eteplirsen, golodirsen, viltolarsen, and casimersen [28,29]. Notably, EMA has so far refused to approve these ASO-therapies based on the same efficacy data [30].

Eteplirsen (Exondys 51™), developed by Sarepta Therapeutics (Cambridge, MA 02142-USA), was the first FDA-approved exon-skipping therapy for DMD, receiving accelerated approval in 2016 [31]. It targets exon 51 and is suitable for approximately 14% of DMD patients with deletions amenable to this exon’s skipping. The drug is a 30-nucleotide PMO (sequence: CTC CAA CAT CAA GGA AGA TGG CAT TTC TAG), administered intravenously at 30 mg/kg once weekly [32]. Eteplirsen’s approval was controversial due to its modest efficacy; dystrophin restoration levels were typically below 1%, with no conclusive evidence of improved motor function. The FDA mandated post-marketing confirmatory trials, which are ongoing, to establish clinical benefit [33].

Golodirsen (Vyondys 53™), also developed by Sarepta, was approved in 2019 for patients with mutations amenable to exon 53 skipping—approximately 8% of DMD cases [34]. It is a 25-nucleotide PMO (sequence: GTT GCC TCC GGT TCT GAA GGT GTTC), dosed at 35 mg/kg weekly via intravenous infusion [35,36]. As part of the accelerated approval process, the FDA mandated that Sarepta Therapeutics conduct clinical trials to verify that golodirsen effectively slows the progression of DMD and enhances motor function. A more extensive clinical trial was recently completed, providing evidence of golodirsen’s long-term safety and therapeutic efficacy [37].

Viltolarsen (Viltepso™), developed by Nippon Shinyaku Pharma and approved by the FDA in 2020, also targets exon 53 and shares a similar mechanism with golodirsen. It is a 21-nucleotide PMO (sequence: CCT CCG GTT CTG AAG GTG TTC) administered at a higher dose—80 mg/kg weekly. Viltolarsen is applicable to 8–10% of DMD patients [38,39]. Unlike earlier PMOs, viltolarsen demonstrated more substantial increases in dystrophin expression, reaching up to 5.9% of normal levels after 25 weeks of treatment. Clinical trial data also showed potential improvement in muscle function, and further studies are underway to confirm these findings [40,41].

Casimersen (Amondys 45™), the most recent PMO approved (2021), also from Sarepta Therapeutics, is indicated for patients with mutations amenable to exon 45 skipping, which applies to around 8–9% of DMD cases. It is a 22-nucleotide PMO (sequence: CAA TGC CAT CCT GGA GTT CCTG), administered weekly at 30 mg/kg via IV infusion. Casimersen works by skipping exon 45 to restore the reading frame and enable the production of a functional dystrophin protein [42,43]. Though early results demonstrated increased dystrophin production, its clinical efficacy is still being assessed in phase III trials. Casimersen, like other ASOs, was approved based on its molecular mechanism rather than definitive improvements in motor outcomes [42,43].

While ASO therapy represents a major advancement in precision medicine for DMD, the field still faces critical challenges. The ASO-mediated exon-skipping strategy is not curative; it does not regenerate lost muscle tissue but aims to slow disease progression. It is mutation-specific, meaning each ASO only benefits a subset of patients. For example, skipping exon 51 benefits ~14%, exon 53 ~8–10%, and exon 45 ~8–9%. This therapeutic precision is both a strength and a limitation, as ASOs must be tailored to individual genotypes [44,45]. ASO therapies typically lead to low levels of dystrophin restoration (often <5%). Although these truncated proteins are functional, their effectiveness is significantly lower than normal dystrophin. Additionally, the cost, weekly IV infusions, and lifelong treatment requirements pose logistical and financial challenges. Nevertheless, with continued research and innovation, ASO-based therapies offer hope for converting the severe DMD phenotype into a milder, BMD-like condition, thereby improving quality of life and life expectancy for affected individuals. Ongoing trials continue to evaluate the clinical benefits of current ASOs, and additional PMO-based candidates are under development. Moreover, efforts are underway to improve ASO chemistry for better cellular uptake, enhanced target binding, and increased exon-skipping efficiency [44,45].

### 2.4. Gene Therapy

Gene therapy for Duchenne muscular dystrophy (DMD) aims to restore dystrophin by providing a functional copy of the DMD gene. This gene addition therapy involves delivering a cDNA copy of functional DMD to target tissues using viral vectors. While most viruses lack a natural affinity for skeletal muscle and cardiac tissue, adeno-associated viruses (AAVs) are a notable exception due to their ability to efficiently infect these tissues. However, a major challenge is the large size of the full-length dystrophin cDNA (~11.4 kb), which far exceeds the AAV vector’s limited packaging capacity (~4.7 kb). To overcome this, researchers have developed truncated versions of dystrophin known as mini-dystrophin and micro-dystrophin constructs, since deleted forms of dystrophin can retain partial functionality, as demonstrated in individuals with BMD. These constructs retain only the most essential domains of the protein, including the N-terminal actin-binding domain (ABD), several spectrin-like repeats, hinge regions, and the cysteine-rich (CR) domain. The reduced size of micro-dystrophin cDNA allows it to be packaged within AAV vectors, and its expression has shown therapeutic potential in preclinical mouse and dog models of DMD [46].

Several clinical trials are currently evaluating the systemic delivery of various micro-dystrophin constructs using different AAV serotypes. Early findings show encouraging levels of micro-dystrophin expression in muscle biopsies, with over 80% of muscle fibers expressing the protein and reaching levels above 60%. However, it remains uncertain whether this will effectively slow disease progression. AAV vectors do not integrate into the patient’s genome, so over time, the therapeutic gene might be lost as muscle cells regenerate. It is still unclear how long the effects will last and how effective the micro-dystrophin proteins will be in improving muscle function.

One of the major limitations of gene editing lies in the immunogenicity of both the re-expressed dystrophin protein and the viral vectors. The dystrophin protein, even in the form of micro-dystrophin, might be perceived as unfamiliar by the immune system, prompting the production of antibodies and potentially interfering with the long-term success of the treatment. Likewise, the immune system may identify the AAV vector as a foreign invader and react against it, reducing the effectiveness of the therapy or triggering harmful side effects. To reduce the risk of immune reactions, participants in clinical trials were screened in advance to ensure they had no pre-existing antibodies against the viral vector, and they received high-dose steroids before treatment. Despite these precautions, treatment induces anti-AAV neutralizing antibodies, which prevent redosing and exclude patients with pre-existing immunity [47]. Researchers are exploring solutions to this issue, including the use of different AAV serotypes, immunosuppressive medications, or antibody-removal techniques like plasmapheresis.

Despite these challenges, gene therapy represents a major leap forward in the treatment of DMD and holds significant potential to alter the disease course in young patients. One of the most significant developments in this field is the FDA’s June 2023 accelerated approval of delandistrogene moxeparvovec (Elevidys^®^), the first AAV-based gene therapy approved in the U.S. for the treatment of DMD. This therapy is indicated for ambulatory pediatric patients aged 4 to 5 years with a confirmed mutation in the DMD gene. Developed by Sarepta Therapeutics, delandistrogene moxeparvovec delivers a gene encoding a micro-dystrophin protein—a shortened 138 kDa version of the normal 427 kDa dystrophin—via a single intravenous infusion at a recommended dose of 1.33 × 10^14^ vg/kg [48,49]. The FDA’s accelerated approval was based on data from a small phase II clinical trial involving 40 boys aged 4–7 years. In this study, 20 children received delandistrogene moxeparvovec and 20 received placebo. After 48 weeks, treated patients demonstrated a mean micro-dystrophin expression of 28% on western blot. Although the primary functional endpoint (North Star Ambulatory Assessment (NSAA)) did not show significant improvement across the entire group, a prespecified subgroup analysis revealed meaningful benefit in the 4–5-year-old cohort, supporting the conditional approval [48,49,50]. In a more recent development, the FDA has expanded the indication for Elevidys, granting traditional approval for ambulatory patients aged 4 years and older and accelerated approval for non-ambulatory patients within the same age group [51]. However, this decision has sparked debate within the medical and regulatory communities due to the limited scope of clinical evidence and the conditional nature of the original approval [52].

### 2.5. Gene Editing Therapy

The advent of genome editing technologies, particularly Clustered Regularly Interspaced Short Palindromic Repeats (CRISPR)/Cas9, has revolutionized the landscape of genetic medicine and opened new possibilities for treating monogenic disorders such as DMD. CRISPR/Cas9 enables targeted modifications to the genome by using guide RNAs (gRNAs) to direct the Cas9 enzyme to specific DNA sequences, where it introduces double-stranded breaks (DSBs). These breaks are repaired by the cell’s own DNA repair mechanisms: either through error-prone non-homologous end joining (NHEJ), particularly relevant in non-dividing cells like muscle tissue, or the more precise homologous recombination pathway, active in dividing cells [53]. In DMD, where affected tissues are largely post-mitotic, gene editing strategies have focused primarily on leveraging NHEJ to restore the reading frame of the dystrophin gene by deleting specific exons, abolishing splice sites, or reframing mutated regions. Notably, gene editing for DMD remains mutation-specific, as different patients require tailored exon targeting.

Several studies in both cell and animal models have shown promising results, with successful dystrophin restoration and improved muscle function [54,55,56]. Notably, editing of muscle stem cells in mouse models has also been demonstrated, offering hope for long-term regenerative potential [57,58]. CRISPR/Cas9 gene editing offers a potentially permanent treatment for Duchenne muscular dystrophy (DMD) by directly correcting the underlying genetic mutation. This approach stands out from current FDA-approved therapies, such as exon-skipping drugs like eteplirsen (Exondys 51), which cost over $1 million per patient per year, and micro-dystrophin gene therapy like delandistrogene moxeparvovec (Elevidys), priced at $3.2 million for a single dose. These high costs make such treatments largely inaccessible in many parts of the world, especially in developing countries. In contrast, CRISPR-based therapies aim to definitely restore dystrophin expression through a single intervention, potentially eliminating the need for lifelong treatment and its associated financial burden [59].

Despite promising preclinical data, the translation of CRISPR-based therapies to clinical practice faces several critical challenges. Chief among these is the efficient and safe delivery of gene editing components to all affected muscle tissues, including the diaphragm and the heart. Adeno-associated virus (AAV) vectors, the most commonly used delivery platform due to their tissue tropism and low immunogenicity, face limitations related to packaging size and potential immune responses. Efforts to overcome these include the use of smaller Cas proteins, dual-vector systems, lipid nanoparticles, exosome-based systems, and engineered AAVs [60].

The potential of ex vivo gene editing is also being investigated [61]. In this approach, patient-derived cells, such as induced pluripotent stem cells (iPSCs), are corrected outside the body and then reintroduced. iPSCs can be differentiated into myogenic precursors and transplanted into the patient to participate in muscle regeneration. However, challenges remain in delivering these cells effectively to muscle tissue and ensuring their long-term survival and integration.

Clinical translation of CRISPR therapies has made significant progress in hematological disorders, with the recent FDA approval of Casgevy, the first CRISPR-based drug for sickle cell disease and transfusion dependent β-thalassemia [62]. This success demonstrates the feasibility and safety of ex vivo CRISPR editing in hematopoietic stem cells, setting the stage for broader clinical applications. In contrast, neuromuscular disorders such as DMD face distinct hurdles, particularly in vivo delivery and immunological risks. The first CRISPR-based clinical trial for DMD (NCT05514249), conducted by Cure Rare Disease, involved a single patient with an exon 1 deletion. The trial utilized AAV9 to deliver dCas9-VP64, aiming to upregulate dystrophin expression. Tragically, the patient experienced acute cardiac and respiratory complications attributed to the high dose of AAV, not the gene editing itself [63]. This event underscores the risks of systemic AAV delivery and highlights the need for safer delivery systems and dosing strategies.

While gene editing offers the promise of a one-time, curative intervention, safety concerns remain. Cas9-induced DSBs can lead to large insertions/deletions, chromosomal rearrangements, or chromothripsis, raising the specter of long-term genotoxicity [64]. Off-target editing and immune responses to Cas9 further complicate the clinical translation. The development of high-fidelity Cas9 variants [65] and optimized gRNAs [66] has mitigated some of these risks, but more work is needed to ensure long-term safety and efficacy.

### 2.6. HDAC Inhibitor Therapy

One of the key pathological features of DMD is the abnormal and sustained activation of histone deacetylases (HDACs), enzymes that remove acetyl groups from histones and other proteins, leading to chromatin compaction and repression of gene transcription. This persistent HDAC activity in DMD disrupts the normal transcriptional programs necessary for muscle regeneration. It also contributes to immune dysregulation, drives fibro-adipogenic progenitor cells (FAPs) toward producing excess connective and fat tissue, and impairs the function of satellite cells, the muscle stem cells responsible for repair. As a result, muscle regeneration fails, and tissue damage accumulates over time [67,68,69]. HDAC inhibition has therefore been explored as a treatment strategy for muscular dystrophies with the potential to work regardless of the specific genetic mutations involved, making it a promising option for all patients with DMD [67,68,69,70,71,72,73,74,75,76,77,78,79].

Givinostat works by inhibiting HDACs, thereby rebalancing the epigenetic environment within muscle tissue. This allows genes involved in muscle repair to be reactivated. In preclinical studies using the mdx mouse model of DMD, givinostat improved muscle histology and performance by reducing fibrosis and inflammation, enhancing muscle regeneration, and increasing the size and function of muscle fibers [76]. The treatment also normalized the expression of certain microRNAs (miRNAs) involved in muscle differentiation and maintenance, which are typically dysregulated in DMD [74].

Clinical data further support givinostat’s therapeutic potential [77,78]. A phase 3 randomized, double-blind, placebo-controlled trial conducted in ambulant boys with DMD showed that givinostat, when used alongside standard corticosteroids, significantly slowed disease progression over an 18-month period. The treatment led to improvements in motor function, muscle strength, and physical performance [78]. Muscle imaging revealed reduced fat infiltration in key muscle groups, consistent with the histological benefits observed in earlier preclinical and phase 2 trials [76,77,78]. The safety profile was favorable, with side effects such as gastrointestinal symptoms and changes in blood parameters being mild to moderate and manageable through dose adjustments [78].

Mechanistically, givinostat exerts its benefits by addressing multiple components of DMD pathology simultaneously. It dampens the chronic inflammatory state in muscle by promoting a shift in immune cell activity from a pro-inflammatory to a more regenerative profile. It also restores the capacity of satellite cells to differentiate into muscle fibers, while preventing FAPs from becoming fibrotic or adipogenic. These combined effects lead to improved muscle quality, preserved tissue architecture, and enhanced repair capacity [68,69,70,71,72,73,74,75,76,77,78].

In March 2024, the FDA approved givinostat (Duvyzat™) for the treatment of DMD in patients aged 6 years and older, representing a significant advancement in the field [79]. Importantly, givinostat is effective regardless of the specific mutation in the DMD gene, making it the first nonsteroidal treatment for DMD approved for broad use.

**Table 1:** FDA/EMA-approved treatments for DMD.

Brand Name	Active Ingredient	Manufacturer	Therapy Type	Target Patients	Approval Year	FDA/EMA	Administration	Side Effects	Additional Notes	References
**Emflaza^®^**	Deflazacort	PTC Therapeutics	Glucocorticoid (Steroid)	≥2 years old patients	2016	Yes/no	Oral	Weight gain, Cushingoid appearance, behavioral changes, hypertension, bone fragility, growth delay	The first FDA-approved corticosteroid treatment for DMD	[17,18,19]
**AGAMREE^®^**	Vamorolone	Santhera Pharmaceuticals	Dissociative steroid	≥2 years old patients	2023	Yes/yes	Oral	Mild GI symptoms, increased appetite, mild weight gain; fewer steroid-like side effects	The only approved medication for DMD in the European Union and the first DMD treatment approved in both the U.S. and EU	[20,21,22]
**Translarna™**	Ataluren	PTC Therapeutics	Protein restoration therapy	≥2 years old ambulatory patients	-	No/non-renewal	Oral	Headache, vomiting, diarrhea, flatulence, increased creatinine phosphokinase	Applies to DMD caused by nonsense mutations by inducing ribosomal readthrough	[23,24,25,26,27]
**Exondys 51™**	Eteplirsen	Sarepta Therapeutics	Exon-skipping (exon 51)	Patients with mutations amenable to exon 51 skipping	2016	Yes/no	Weekly IV infusion	Balance disorder, vomiting, possible renal toxicity (kidney monitoring recommended)	First exon-skipping therapy approved for DMD; applies to 14% of DMD patients	[31,32,33]
**Vyondys 53™**	Golodirsen	Sarepta Therapeutics	Exon-skipping (exon 53)	Patients with mutations amenable to exon 53 skipping	2019	Yes/no	Weekly IV infusion	Headache, fever, cough, vomiting, risk of kidney injury (kidney monitoring recommended)	Applies to 8–10% of DMD patients	[34,35,36,37]
**Viltepso™**	Viltolarsen	NS Pharma	Exon-skipping (exon 53)	Patients with mutations amenable to exon 53 skipping	2020	Yes/no	Weekly IV infusion	Upper respiratory infections, injection site reactions, proteinuria (kidney monitoring recommended)	Applies to 8–10% of DMD patients	[38,39,40,41]
**Amondys 45™**	Casimersen	Sarepta Therapeutics	Exon-skipping (exon 45)	Patients with mutations amenable to exon 45 skipping	2021	Yes/no	Weekly IV infusion	Headache, fever, increased liver enzymes, possible renal toxicity (kidney monitoring recommended)	Applies to 8–9% of DMD patients	[42,43]
**Elevidys^®^**	Delandistrogene moxeparvovec	Sarepta Therapeutics	Gene therapy (micro-dystrophin)	≥4 years old 4 ambulatory and non-ambulatory patients	2023	Yes/no	Single IV infusion	Vomiting, fever, liver enzyme elevation, immune reaction (requires steroid prophylaxis)	One-time gene therapy	[48,49,50,51,52]
**Duvyzat™**	Givinostat	Italfarmaco S.p.A.	HDAC inhibitor (epigenetic)	≥6 years old with any dystrophin mutation	2024	Yes/no	Oral	GI disturbances, thrombocytopenia, elevated creatine kinase, fatigue	First nonsteroidal treatment for DMD approved for broad use; may be used alongside other therapies	[74,75,76,77,78,79]

### 2.7. Future Directions for Combination Therapies

With the advent of new drugs now approved for DMD treatment there is potential to explore their combined use. The rationale behind combining treatments is that therapies aimed at restoring dystrophin depend on the amount and quality of muscle tissue enhanced by other therapies, like givinostat, aimed to counteract the secondary effects of the pathology.

For instance, micro-dystrophin provided by gene therapy (delandistrogene moxeparvovec) is not fully functional, it may only slow disease progression rather than halt it entirely. Therefore, pairing it with a second therapy that further protects muscle tissue, like vamorolone and givinostat, could provide additional benefit.

Exon-skipping therapies (such as eteplirsen, golodirsen, casimersen, and viltolarsen) use antisense oligonucleotides to modify the splicing of dystrophin pre-mRNA. However, dystrophin transcript levels are notably reduced in DMD patients due to chromatin remodeling. Givinostat has the potential to enhance the expression of dystrophin transcripts and may offer an extra advantage when used in conjunction with exon skipping, boosting their effectiveness. Indeed, studies using the mdx mouse model demonstrated that combining givinostat with exon-skipping agents led to higher levels of both dystrophin mRNA and protein compared to exon skipping alone [80].

Finally, givinostat and vamorolone have complementary mechanisms that may offer synergistic benefits in treating DMD. Givinostat improves muscle regeneration and reduces inflammation and fibrosis through epigenetic modulation, while vamorolone provides anti-inflammatory effects with a better safety profile than traditional corticosteroids. Used together, they could enhance muscle quality, reduce damage, and slow disease progression more effectively than either treatment alone.

## 3. Diagnostic Approaches in DMD

**Figure 2:**
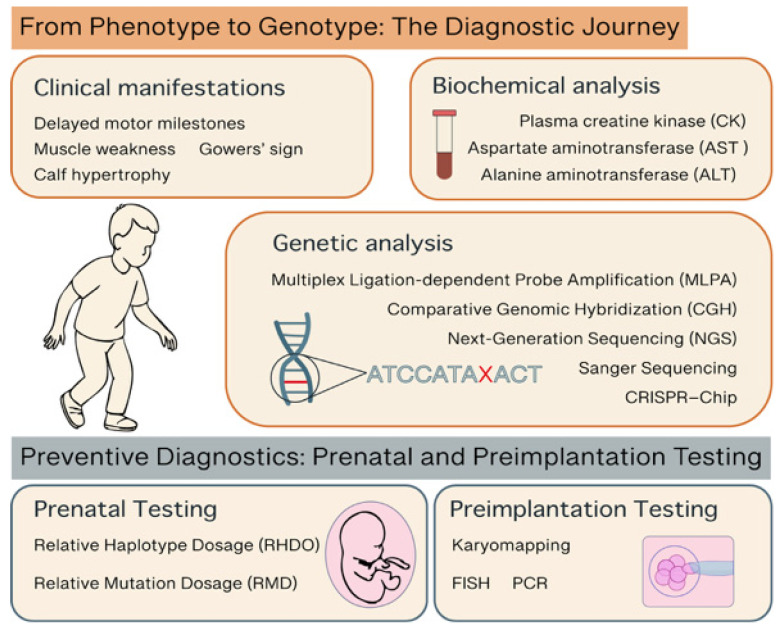
Representative picture of the diagnostic steps and methods for DMD.

Early diagnosis of DMD is essential for timely intervention, optimal clinical management, and informed genetic counseling. As a progressive and irreversible neuromuscular disorder, early identification of DMD allows for the prompt initiation of supportive therapies, such as corticosteroids, HDAC inhibitors and multidisciplinary care, which can significantly delay disease progression and improve quality of life. Moreover, early diagnosis enables families to access genetic counseling and reproductive options, including prenatal and preimplantation testing. With the emergence of novel therapies, such as exon-skipping and gene therapy, establishing a diagnosis during the presymptomatic phase may become increasingly critical to maximize therapeutic efficacy. Thus, enhancing awareness and improving access to early diagnostic pathways, including genetic testing and newborn screening, represent key priorities in the global effort to combat DMD (Figure 2, Table 2).

### 3.1. From Phenotype to Genotype: The Diagnostic Journey

Duchenne muscular dystrophy (DMD) should be suspected in young boys between the ages of 2 and 4 who present with delayed motor milestones, muscle weakness, calf hypertrophy, and the Gowers’ sign—a clinical feature where a child uses their hands to climb up their legs when rising from the floor, due to weakness in the upper leg and hip muscles. Children with DMD often develop a waddling gait, frequent falls, and may walk on their toes due to tight calf muscles. As the disease progresses, loss of ambulation typically occurs by the early teens. Additionally, DMD patients may show cognitive impairment at the time of diagnosis, and speech delay is common [81]. These neurological features should raise clinical suspicion for DMD, especially when presenting alongside developmental delays, behavioral or emotional difficulties, autism spectrum disorder, or attention-deficit/hyperactivity disorder. In fact, up to 72.7% of individuals with DMD show at least one of these symptoms [82]. The most common neurodevelopmental and behavioral features include emotional or behavioral dysregulation (38.7%), inattention or hyperactivity (31.4%), obsessive-compulsive traits (25.0%), and language or speech delays (24.4%). These symptoms are important comorbidities in DMD and appear to be influenced by genotype. Mutations located near the 3′ end of the DMD gene, particularly between exons 31 and 79, are associated with a higher risk of inattention, speech and language delays, and global intellectual impairment, as well as a greater likelihood of multiple co-occurring neurodevelopmental symptoms compared to mutations located upstream of exon 30 [82].

The initial diagnostic step involves measuring plasma creatine kinase (CK) levels, as these are typically markedly elevated from birth in individuals with DMD, with values often exceeding 20,000 U/L. Plasma levels of liver enzymes such as AST and ALT are also increased due to muscle damage, although CK is a more specific biomarker for muscle injury and therefore preferred in the diagnostic process [81]. While elevated CK levels support the suspicion of DMD, they are not sufficient for a definitive diagnosis, since CK elevation can also occur due to other muscle disorders, injuries, or intense physical activity [81,83]. Therefore, while plasma CK testing is useful for identifying dystrophin-related conditions in symptomatic individuals, it is not specific to DMD.

Measuring plasma CK levels from dried blood spots is a practical approach that can be employed also for newborn screening of DMD. A systematic review of 11 studies found that CK testing in newborns is effective in identifying patients with true DMD, demonstrating high specificity (≥90%) and sensitivity (≥80%). The rate of false negatives was reported to be very low, which makes this test particularly valuable for diagnostic screening. Additionally, the test shows a strong likelihood of confirming the presence of the disease and a reasonable likelihood of ruling it out [84].

Confirming the presence of a DMD gene mutation is essential for an accurate and definitive diagnosis. Genetic confirmation is also important for initiating multidisciplinary care, identifying potential carriers in the family, offering genetic counseling, and determining whether the patient is eligible for currently approved mutation-specific therapies [83]. Once a pathogenic mutation has been confirmed in a patient, testing the mother to determine carrier status is recommended. If the mother is a carrier, further family testing and counseling are necessary, as her female relatives may also carry the mutation. Carrier mothers have a 50% chance of having another affected son or a carrier daughter [83].

Large deletions or duplications in the DMD gene are typically easier to detect using methods such as multiplex ligation-dependent probe amplification (MLPA) or comparative genomic hybridization (CGH). In contrast, identifying single nucleotide variants or small insertions and deletions often requires more precise techniques like Sanger sequencing or next-generation sequencing (NGS) [2,83]. Since about 70% of DMD cases involve deletions or duplications affecting one or more exons of the dystrophin gene, MLPA remains a commonly used initial diagnostic test in many countries. However, recent advancements have made NGS more widely available and accessible, allowing for quicker and more definitive diagnoses. NGS has improved the diagnostic process for dystrophinopathies by offering a high-throughput and precise method for identifying a broad spectrum of mutations, including large deletions, duplications, and point mutations, within the DMD gene [2,81,83]. Additionally, NGS panels can include other genes associated with muscular dystrophies, helping to distinguish DMD and BMD dystrophinopathies from limb-girdle muscular dystrophies or congenital myopathies that may present similarly in early life. These disorders often share overlapping features such as muscle weakness, elevated serum CK levels, and progressive motor decline, making early differential diagnosis particularly challenging when muscle atrophy is the only presenting sign. High precision in molecular diagnosis not only facilitates accurate classification and prognosis but is also increasingly important for guiding eligibility for emerging gene-targeted therapies.

Although methods for detecting nucleic acid mutations have been refined over the past 30 years, they remain time-consuming and expensive. These technologies rely on complex, multi-step processes that require numerous reagents, specialized equipment, and trained personnel. As a result, there is a growing need for new, cost-effective, and compact diagnostic tools that simplify nucleic acid detection and broaden its clinical accessibility. In recent studies, CRISPR-Cas9-based methodologies have been utilized to offer new possibilities for fast, accurate, and accessible genomic testing [85,86,87]. Of note is the development of CRISPR–Chip, an innovative diagnostic platform that combines CRISPR–Cas9 technology with a graphene-based field-effect transistor (gFET) to detect specific DNA sequences directly from unamplified genomic samples [87]. CRISPR–Chip employs a catalytically inactive Cas9 protein that is complexed with a programmable single-guide RNA. This complex is immobilized on the graphene surface of the transistor. When the CRISPR complex encounters its complementary DNA target within the intact genome, it binds to it, changing the electrical properties of the graphene. These changes can then be read by a simple handheld electronic reader, making the process label-free, rapid (within 15 min), and highly sensitive [87]. CRISPR–Chip may bypass the need for sequence amplification for hereditary disease analysis as the genomic material required for CRISPR–Chip analysis is obtainable via commercially available buccal swab methods.

The biosensor was successfully tested with clinical samples from patients with DMD, detecting common exon deletions without pre-processing or amplification [87]. While this proof-of-concept focused on two common mutations, the technology is easily programmable to target other genomic regions by simply modifying the sgRNA sequence, and future improvements may enable it to detect single-nucleotide polymorphisms and expand its clinical applications. CRISPR–Chip holds significant promise as a point-of-care diagnostic tool and as a platform for digital genomics and personalized medicine.

When interpreting genetic test results in patients with suspected dystrophinopathies, it is essential to consider the clinical presentation alongside the molecular findings. In more than 90% of cases, out-of-frame mutations are associated with DMD, while in-frame mutations typically correlate with BMD [1,2]. However, exceptions to this genotype–phenotype correlation do occur. For instance, if a patient presents with clinical features consistent with DMD despite having an in-frame mutation, a muscle biopsy may be considered to evaluate dystrophin protein expression. This additional analysis can help determine whether alternative splicing or other molecular mechanisms are responsible for the observed discrepancy. In general, a muscle biopsy is not required to confirm a diagnosis of DMD, as genetic testing is usually definitive. However, in cases where no mutation is identified, biopsy may be necessary to assess the presence and quality of dystrophin using immunofluorescence or Western blot techniques. An absence of dystrophin supports a diagnosis of DMD, whereas reduced levels or an abnormal molecular weight may indicate BMD [2,81].

### 3.2. Preventive Diagnostics: Prenatal and Preimplantation Testing

In female carriers, who may be asymptomatic or present with mild symptoms, MLPA plays a crucial role in identifying heterozygous mutations. Its high sensitivity and ability to quantify exon copy number make it especially valuable for confirming carrier status in at-risk individuals and for genetic counseling in families affected by DMD [2,83]. Once a disease-causing mutation in the DMD gene is confirmed in an affected family member, prenatal testing and preimplantation genetic testing are possible to avoid inheritance.

Currently, prenatal diagnosis for pregnancies at risk of Duchenne or Becker muscular dystrophy (DMD/BMD) typically involves non-invasive fetal sexing followed by invasive procedures such as chorionic villus sampling (CVS) or amniocentesis if the fetus is male [88]. These invasive tests carry a risk of miscarriage ranging from 0.5% to 1%, and no alternative exists for women who choose to avoid them [89]. The identification of cell-free fetal DNA (cffDNA) in maternal plasma in 1997 led to major advancements in prenatal diagnostics and opened the way for the development of non-invasive prenatal testing (NIPT), initially used for detecting fetal aneuploidies and more recently expanded to include single-gene disorders (SGDs), like DMD and BMD, offering a safer and earlier testing option [90].

However, diagnosing DMD through non-invasive methods presents unique challenges due to the high levels of maternal mutant alleles in circulation, which can overshadow the relatively small proportion—about 10%—of fetal DNA in maternal blood. Technological advances, particularly in massively parallel sequencing (MPS), have allowed for the detection of fetal aneuploidies and facilitated progress in the development of NIPT for SGDs [91,92]. New molecular techniques have been developed to assess the relative quantity of mutant and wild-type alleles, improving the accuracy of fetal mutation detection even in the presence of excess maternal DNA [93]. These tests are primarily intended for pregnancies with a known family history of DMD and are often considered diagnostic, eliminating the need for invasive confirmation. Despite their promise, concerns remain regarding clinical validity, cost-effectiveness, and ethical considerations, especially given the low prevalence of DMD, which makes widespread validation difficult. The absence of formal clinical guidelines and increasing commercialization of these tests to unselected populations raise additional ethical concerns, including inadequate genetic counseling, pressure to undergo testing, and decisions related to pregnancy termination.

There are two primary molecular approaches for non-invasive DMD detection: Relative Haplotype Dosage (RHDO) and Relative Mutation Dosage (RMD) [93]. RHDO analyzes the distribution of maternal haplotypes associated with mutant and wild-type alleles in cffDNA. While it allows for testing without knowing the exact mutation, it often requires DNA from both parents and a previously affected child, which may not be available in first pregnancies. It is less suitable for detecting de novo mutations or maternal germline mosaicism and is influenced by recombination events in the DMD gene, which can affect accuracy. In contrast, RMD directly calculates the ratio of mutant to wild-type alleles using family-specific probes, making it more suitable for identifying de novo mutations and maternal mosaicism, and eliminating the need for haplotype construction or recombination analysis. However, RMD is not well suited for detecting large deletions or duplications in the DMD gene [93]. Although these methods are technically complex and costly, they offer significant advantages, including earlier detection, no risk of miscarriage, and reduced anxiety for families at risk. To ensure their ethical and effective use, NIPT for DMD should be implemented within specialized programs that include comprehensive genetic counseling and emphasize informed decision-making.

For couples at risk of having a child with Duchenne muscular dystrophy (DMD), preimplantation genetic diagnosis (PGD) through in vitro fertilization (IVF) offers another option to select and implant only unaffected embryos, avoiding the need for prenatal diagnosis and possible pregnancy termination [94]. PGD for DMD is technically complex due to the large size and variability of the dystrophin gene. Traditional methods like PCR and FISH require custom protocols for each family, making them time-consuming and expensive [95,96,97]. An advanced alternative, karyomapping, allows for faster and broader genetic analysis, including both mutation detection and chromosome balance, though it also has limitations like cost and occasional ambiguous results [98]. While PGD offers the significant advantage of preventing the transmission of the disorder before pregnancy is established, it also presents several limitations. The success rate of achieving a pregnancy through IVF and PGD is modest and varies across individuals. The process is emotionally, financially, and physically demanding, often involving repeated cycles, hormone treatments, and uncertainty [94]. As a result, PGD is mostly used by couples who already require IVF to conceive. For others, its use remains limited due to the high cost and complexity of the procedures.

In conclusion, both PGD and NIPT offer valuable options for managing the risk of DMD, each with its own benefits and limitations. Proper implementation depends on expert genetic counseling, access to advanced technologies, and careful ethical consideration.

**Table 2:** Diagnostic methods for DMD.

Diagnostic Method	Invasiveness	Purpose	What It Detects	When It Is Used	Notes	References
**Creatine Kinase (CK) Test**	Non-invasive	Initial screening	Elevated CK (>10*×* normal) suggests muscle damage	First step in suspected DMD	High CK is common but not specific to DMD	[81,83,84]
**Multiplex Ligation-dependent Probe Amplification (MLPA)**	Non-invasive	Definitive diagnosis	Detects large deletions/duplications in the DMD gene	Initial genetic test for diagnosis of common DMD mutations	Cannot detect small mutations	[2,83]
**Next-Generation Sequences (NGS)**	Non-invasive	Definitive diagnosis	Point mutations, deletions, duplications in the DMD gene	Gold standard for diagnosis of all DMD mutations	Most advanced and widely used today.	[2,81,83]
**CRISPR–Chip**	Non-invasive	Definitive diagnosis	Common mutations	Not yet available in clinical practice	Rapid (within 15 min), and bypass sequence amplification.	[85,86,87]
**Muscle Biopsy**	Invasive	Definitive diagnosis	Dystrophin expression via immunostaining	Rarely used today; reserved for unclear cases	Confirms lack or absence of dystrophin protein	[2,81]
**Chorionic villus sampling (CVS)/Amniocentesis**	Invasive	Prenatal Genetic Testing	In families with known DMD mutation	For at-risk families	Requires family history or prior diagnosis	[88,89]
**Relative Haplotype Dosage (RHDO)**	Non-invasive	Prenatal Genetic Testing	Mutation in cell-free fetal DNA	For at-risk families	Not suitable for detecting de novo mutations or maternal germline mosaicism.	[93]
**Relative Mutation Dosage (RMD)**	Non-invasive	Prenatal Genetic Testing	Mutation in cell-free fetal DNA	For at-risk families	Not suitable for detecting large deletions or duplications	[93]
**FISH**	Non-invasive	Preimplantation Testing	Mutation in embryonic cell	For at-risk families using IVF	Requires known familial mutation	[95,96,97]
**PCR**	Non-invasive	Preimplantation Testing	Mutation in embryonic cell	For at-risk families using IVF	Requires known familial mutation	[95,96,97]
**Karyomapping**	Non-invasive	Preimplantation Testing	Mutation in embryonic cell	For at-risk families using IVF	Faster and broader genetic analysis, including both mutation detection and chromosome balance	[98]

## 4. Conclusions

Recent advancements in the treatment and diagnosis of DMD have significantly reshaped the clinical landscape, offering new hope to patients and families. While corticosteroids remain the mainstay of care, emerging therapies—including exon skipping, gene transfer, gene editing, and HDAC inhibition—are beginning to address the root causes and multiple consequences of the disease. Simultaneously, the development of advanced diagnostic tools—from next-generation sequencing to CRISPR-based biosensors—has enhanced the speed and precision of diagnosis. Looking ahead, the combination of early, accessible diagnostics with tailored therapeutic regimens holds great promise for improving outcomes. The future of DMD care lies in the integration of these therapeutic innovations with individualized diagnostics, ultimately moving toward personalized, multi-modal treatment strategies that may one day transform DMD from a fatal condition into a manageable disease.

## Data Availability

Data sharing does not apply to this article as no datasets were generated.

## Abbreviations

The following abbreviations are used in this manuscript:

DMD	Duchenne muscular dystrophy
BMD	Becker muscular dystrophy
HDAC	histone deacetylase
DAPC	dystrophin-associated protein complex
CK	creatine kinase
nNOS	neuronal nitric oxide synthase
FDA	Food and Drug Administration
EMA	European Medicines Agency
CHMP	Committee for Medicinal Products for Human Use
ASO	antisense oligonucleotides
PMO	phosphorodiamidate morpholino oligomer
AAV	adeno-associated virus
CRISPR	Clustered Regularly Interspaced Short Palindromic Repeats
gRNAs	guide RNAs
DSB	double-stranded breaks
NHEJ	non-homologous end joining
iPSC	induced pluripotent stem cells
FAPs	fibro-adipogenic progenitors
AST	aspartate aminotransferase
ALT	alanine aminotransferase
MLPA	multiplex ligation-dependent probe amplification
CGH	comparative genomic hybridization
NGS	next-generation sequencing
cffDNA	cell-free fetal DNA
NIPT	non-invasive prenatal testing
SGD	single-gene disorders
RHDO	relative haplotype dosage
RMD	relative mutation dosage
IVF	in vitro fertilization
PGD	preimplantation genetic diagnosis
FISH	fluorescence in situ hybridization
